# Epidemiology of nontuberculous mycobacterial infections in the U.S. Veterans Health Administration

**DOI:** 10.1371/journal.pone.0197976

**Published:** 2018-06-13

**Authors:** Makoto M. Jones, Kevin L. Winthrop, Scott D. Nelson, Scott L. Duvall, Olga V. Patterson, Kevin E. Nechodom, Kimberly E. Findley, Lewis J. Radonovich, Matthew H. Samore, Kevin P. Fennelly

**Affiliations:** 1 VA Salt Lake City Health Care System, Salt Lake City, Utah, United States of America; 2 Department of Internal Medicine, Division of Epidemiology, University of Utah, Salt Lake City, Utah, United States of America; 3 Public Health and Preventive Medicine, Division of Infectious Diseases, Oregon Health and Sciences University, Portland, Oregon, United States of America; 4 Department of Biomedical Informatics, Vanderbilt University Medical Center, Nashville, Tennessee, United States of America; 5 National Center for Occupational Health and Infection Control, Patient Care Services (Public Health), Veterans Health Administration, Gainesville, Florida, United States of America; 6 Formerly with the National Center for Occupational Health and Infection Control, Patient Care Services (Public Health), Veterans Health Administration, Gainesville, Florida, United States of America; 7 Pulmonary Clinical Medicine Section, National Heart, Lung, and Blood Institute, National Institutes of Health, Bethesda, Maryland, United States of America; National Institute of Infectious Diseases, JAPAN

## Abstract

**Objective:**

We identified patients with non-tuberculous mycobacterial (NTM) disease in the US Veterans Health Administration (VHA), examined the distribution of diseases by NTM species, and explored the association between NTM disease and the frequency of clinic visits and mortality.

**Methods:**

We combined mycobacterial isolate (from natural language processing) with ICD-9-CM diagnoses from VHA data between 2008 and 2012 and then applied modified ATS/IDSA guidelines for NTM diagnosis. We performed validation against a reference standard of chart review. Incidence rates were calculated. Two nested case-control studies (matched by age and location) were used to measure the association between NTM disease and each of 1) the frequency of outpatient clinic visits and 2) mortality, both adjusted by chronic obstructive pulmonary disease (COPD), other structural lung diseases, and immunomodulatory factors.

**Results:**

NTM cases were identified with a sensitivity of 94%, a specificity of >99%. The incidence of NTM was 12.6/100k patient-years. COPD was present in 68% of pulmonary NTM. NTM incidence was highest in the southeastern US. Extra-pulmonary NTM rates increased during the study period. The incidence rate ratio of clinic visits in the first year after diagnosis was 1.3 [95%CI 1.34–1.35]. NTM patients had a hazard ratio of mortality of 1.4 [95%CI 1.1–1.9] in the 6 months after NTM identification compared to controls and 1.99 [95%CI 1.8–2.3] thereafter.

**Conclusions:**

In VHA, pulmonary NTM disease is commonly associated with COPD, with the highest rates in the southeastern US. After adjustment, NTM patients had more clinic visits and greater mortality compared to matched patients.

## Introduction

Nontuberculous mycobacterial (NTM) diseases exhibit regional variation [1–5] but rates appear to be rising. Changing NTM epidemiology has often been noticed when NTM isolates have increased in specimens collected for suspicion of *Mycobacterium tuberculosis* [6, 7] but this is a difficult measure to interpret. Of further concern is that the observed mortality rate attributed to NTM may be rising, although this could be due to aging demographics, a growing population receiving immunomodulatory agents, and rising chronic obstructive pulmonary disease (COPD) [8–10]. Both the ubiquity of NTM in sampled water sources [11] and an increasing seroprevalence of *M*. *avium* complex suggest that increasing environmental exposure may be in play as well [12].

COPD is now the most common underlying disease associated with pulmonary NTM mortality [8]. The recent recommendation to consider prophylactic macrolide monotherapy (macrolides being a mainstay of NTM therapy) for severe COPD patients [13] may have implications for those who eventually develop NTM disease as it may induce acquired drug resistance to macrolides in NTM [14]. Thus, a better understanding of the epidemiology of NTM in COPD patients is needed to guide policies and practices.

The United States (U.S.) Veterans Health Administration (VHA) is the largest integrated national health care system in the U.S., and there is a high prevalence of COPD among its patients [15, 16]. We hypothesized that COPD is a common co-morbid condition [17] in VA patients diagnosed with NTM lung disease, and that NTM would be associated with more healthcare utilization and higher mortality.

## Methods

This study was approved by institutional review boards at the University of Utah and the University of Florida, and the Research and Development (R&D) Committees at the VA Salt Lake City Health Care System and the VA North Florida/South Georgia Veterans Health System. Informed consent was waived because this large, minimal risk study of retrospective data and no patient contact could not be performed if individual consent was performed. Data from January 2008 through December 2012 were extracted from the VA Corporate Data Warehouse and made accessible for research through the Veterans Informatics and Computing Infrastructure. The identification of physician diagnoses for NTM infections were based on one or more ICD-9 codes of 031, excluding 031.8 and 031.9 as they were not specific for NTM infection (these codes are present in approximately half but are present alone in ~40% of all 031 encounters). In- and outpatient ICD9-CM diagnoses, as well as CPT codes were used to identify COPD, bronchiectasis, cancer, other pulmonary diagnoses, HIV, and receipt of disease-modifying antirheumatic drugs (DMARD). (Appendix)

Natural language processing (NLP) was used to identify mycobacterial species from clinical isolates recorded in free-text microbiology record data [18]. To cover all mycobacterial testing in VHA, laboratory data were also included. We included all NTM species found in microbiology records except for *M*. *gordonae*, which is nearly always a contaminant [19]. We then grouped the major species. *M*. *avium*, *M*. *intracellulare*, and *M*. *avium-intracellulare* group were classified as *M*. *avium* complex (MAC). *M*. *abscessus* and *M*. *chelonae* were combined to form the *M*. *abscessus-chelonae* group. Species belonging to *M*. *fortuitum and M*. *terrae complexes* were grouped at the organism complex level. SNOMED CT was used for classification.

**Fig 1:**
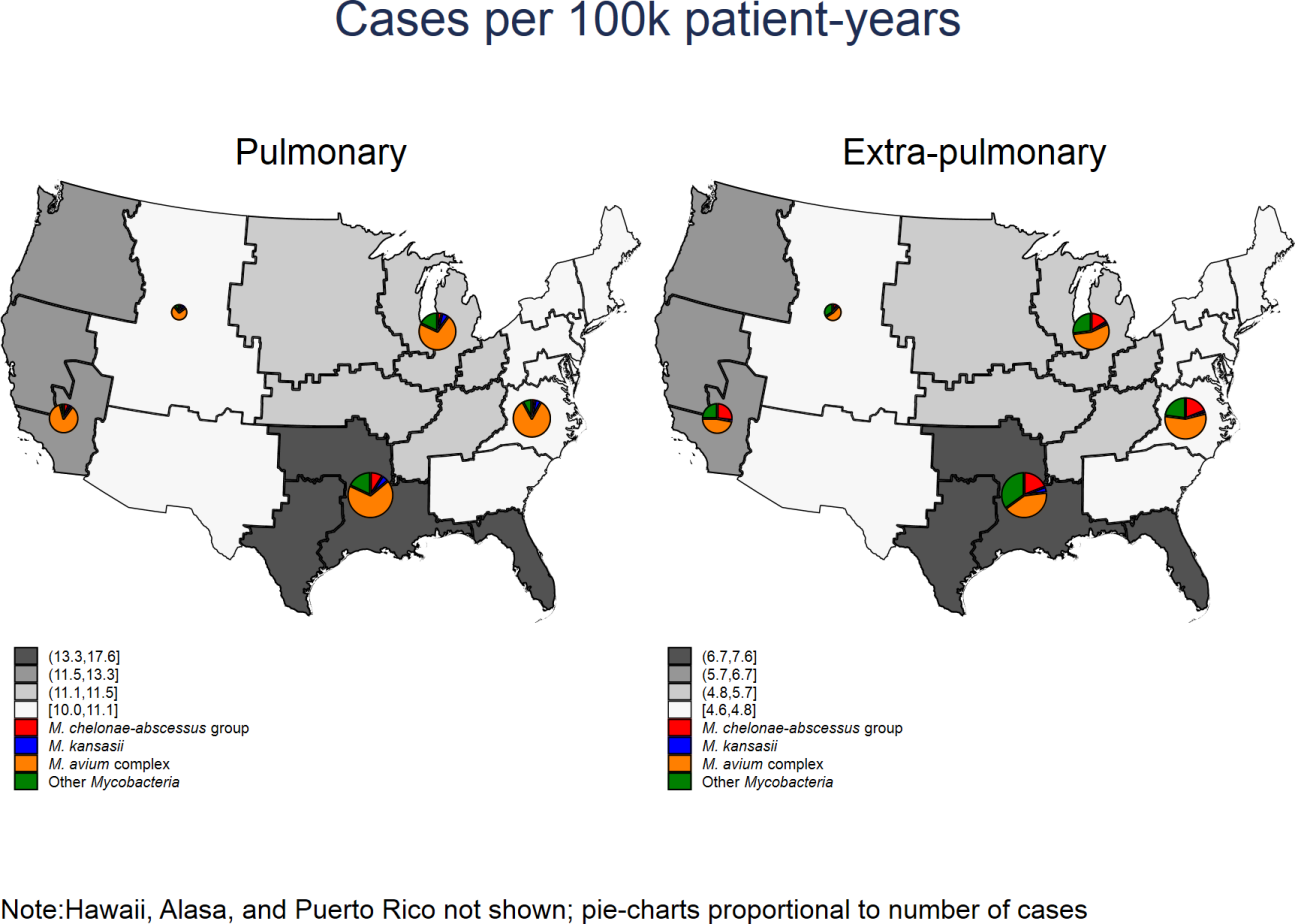
Incident rates of NTM cases per 100,000 patient-years by region and by microbiologically-identified organism from 2009 through 2012. Reprinted from Esri^®^ ArcGIS Online^®^ under a CC BY license, with permission from Environmental Systems Research Institute, Inc., original copyright 2017.

The locations from which isolates were collected were classified geographically by Veterans Integrated Service Networks (VISNs): Atlantic (VISNs 1–7), Gulf Coast (VISNs 8, 16, 17), Midwest (VISNs 9–12, 15, and 23), Mountain (VISNs 18 and 19), and Pacific (VISNs 20, 21, and 22). (Fig 1)

We used an electronic case definition to approximate the microbiological diagnostic criteria outlined in guidelines from 2007 [19]; clinical criteria were not assessed electronically. For pulmonary NTM, we stipulated either 1) the isolation of the same NTM species from at least two sputa, a specific mention of one NTM species coupled with a second culture compatible with the first (e.g., #1: *Mycobacterium avium*, #2: *positive for AFB*), or 2) one NTM-positive bronchoalveolar lavage. We also identified cases for which an ICD-9-CM code was present for pulmonary NTM (031.0). For extra-pulmonary NTM disease, the microbiology criterion was at least one positive culture of an NTM species from one normally sterile, non-pulmonary site (e.g., includes tissue, cerebrospinal fluid but excludes skin or wound swabs). We also identified cases for which an ICD-9-CM code was present for extra-pulmonary NTM disease (all other included NTM infection codes) and for which no pulmonary NTM codes were present. The time of a case was assigned to whichever came first of 1) the specimen collection date of the first test that met microbiology criteria or 2) the date of the first NTM diagnostic code.

To assess the specificity of our electronic case definition, we looked for concordance with at least 30 days of prescription for any of the following possible NTM antimicrobials: levofloxacin, moxifloxacin, tetracyclines, macrolides, linezolid, trimethoprim/sulfamethoxazole, cefoxitin, aminoglycosides, and imipenem. These agents were chosen to minimize the overlap with *M*. *tuberculosis*.

### Pilot study and validation

To validate our electronic case definition against chart review, we manually reviewed 148 patient charts and determined first, whether microbiology data were present and second, whether NTM disease was present according to guidelines (clinical criteria included the presence of symptoms, radiological findings, and the exclusion of other diagnoses) [19]. Because a random sampling from the overall population would sample too few true positives, we took random samples from each of four categories: 1) those with an ICD-9-CM diagnosis of NTM and at least one positive microbiology specimen, 2) those with an ICD-9-CM diagnosis without a positive microbiology specimen, 3) those without an ICD-9-CM diagnosis but with a positive microbiology specimen, and 4) those without either. We accounted for our sampling scheme by using inverse probability weighting to estimate sensitivity, specificity, and predictive values and reported bias-corrected bootstrapped confidence intervals [20, 21].

### Incidence density and period prevalence

Patients were allowed to contribute only one incident case in their lifetime. An incident case was one that met microbiology or diagnosis criteria and was defined to occur at the time of the first of either criterion met. It could not be preceded by any prior positive criteria in VHA patient records. A prevalent case was defined as a case from the time of incidence until death.

Incidence density was calculated by denominating incident cases by the patient years for the population being examined. In turn, patient years were counted as the number of unique patients at a VHA station with one or more in- or outpatient visits during a calendar year; thus, even one visit counted as a full patient year for the calendar year. Patients could only count for the station that they visited most in a year. Ties were broken by taking the first station visited in a year.

Separately, the period prevalence was calculated for calendar year 2012 by denominating the number of unique patients alive with a history of NTM in 2012 by the number of patients alive during all or part of 2012.

### Nested case-control study

To explore the association between NTM disease and healthcare utilization and mortality, we performed a matched, nested case-control study. Cases were defined using the electronic case definition of NTM above. Four controls were matched to each case on 1) age within two years and 2) location where diagnosed, including type of clinic or inpatient location. The first four controls with visit times closest to the case were selected. Reported gender and other known risk factors were included as explanatory variables. We used two different models to investigate the association between NTM disease and two outcomes: the count of outpatient visits over the subsequent year and time to death. Models examining outpatient visits were fit using a Poisson model, accounting for multiple controls. Cox regression was used to estimate the contribution of NTM disease on time to death, stratifying cases with their matched controls. The proportional hazards assumptions was tested using the Grambsch and Therneau test.

#### Other statistical analyses

T-tests and analysis of variance (ANOVA) were used as appropriate. Moran’s I was used to demonstrate clustering manifested by spatial autocorrelation [22]. Positive values up to +1 indicate positive spatial autocorrelation, while negative values indicate negative spatial autocorrelation. Analyses were performed using STATA 12.1.

## Results

The number of Veterans that met inclusion criteria during the study period was 8.2 million. Among them, 6,031 unique individuals revealed some evidence of NTM in either microbiology reports and/or an NTM-related ICD-9-CM code.

By comparing NLP-extracted microbiology data to a reference of manual chart extraction, we measured a sensitivity of 92.9% (95%CI 79.7–100%), specificity of >99.9% (95%CI >99.9%), positive predictive value (PPV) of >99.9% (95%CI >99.9%), and negative predictive value (NPV) of >99.9% (95%CI >99.9%). Table 1 demonstrates the distribution of mycobacterial categories that were identified and met microbiological criteria before collapsing for further analysis.

**Table 1:** Mycobacterial categories found in microbiology and laboratory data in VHA.

Organisms Identified	N	% category	% total
**SLOW GROWERS**			80.97%
*Mycobacterium avium-intracellulare* group	2,546	86.33%	69.91%
*M*. *kansasii*	168	5.70%	4.61%
*M*. *xenopi*	74	2.51%	2.03%
*M*. *terrae* complex	63	2.14%	1.73%
*M*. *simiae*	51	1.73%	1.40%
*M*. *szulgai*	20	0.68%	0.55%
*M*. *scrofulaceum*	11	0.37%	0.30%
*M*. *asiaticum*	8	0.27%	0.22%
*M*. *malmoense*	3	0.10%	0.08%
*M*. *genavense*	2	0.07%	0.05%
*M*. *triplex*	2	0.07%	0.05%
*M*. *haemophilum*	1	0.03%	0.03%
**RAPID GROWERS**			16.80%
*M*. *chelonae-abscessus* group	289	47.22%	7.94%
*M*. *fortuitum* complex	229	37.42%	6.29%
*M*. *mucogenicum*	38	6.21%	1.04%
*M*. *marinum*	28	4.58%	0.77%
*M*. *flavescens*	6	0.98%	0.16%
*M*. *goodii*	4	0.65%	0.11%
*M*. *smegmatis*	4	0.65%	0.11%
Other rapid growing mycobacteria	4	0.65%	0.11%
*M*. *mageritense*	2	0.33%	0.05%
*M*. *wolinskyi*	2	0.33%	0.05%
*M*. *chitae*	1	0.16%	0.03%
*M*. *farcinogenes*	1	0.16%	0.03%
*M*. *neoaurum*	1	0.16%	0.03%
*M*. *peregrinum*	1	0.16%	0.03%
*M*. *porcinum*	1	0.16%	0.03%
*M*. *septicum*	1	0.16%	0.03%
**OTHER**			2.22%
unspecified non-TB *Mycobacterium*	62	76.54%	1.70%
Scotochromogenic mycobacteria	7	8.64%	0.19%
*M*. *interjectum*	6	7.41%	0.16%
*M*. *lentiflavum*	5	6.17%	0.14%
*M*. *moriokaense*	1	1.23%	0.03%

After assigning all isolates to one of four major mycobacteriological categories over the entire 5-year period, there were 3,566 NTM cases identified by microbiological criteria and 3,968 cases identified by ICD-9-CM code with only 24.2% overlap. Of cases meeting microbiological criteria, 2,960 cases (83%) met criteria for pulmonary NTM and 606 cases (17%) for extra-pulmonary NTM. Of pulmonary cases, 75.8% were *M*. *avium* complex, followed by *M*. *chelonae-abscessus* complex (5.8%), and *M*. *kansasii* (5.2%). The remainder was composed of 26 other species and three unspeciated groups. The distribution of the same pathogens in extra-pulmonary cases was 50.0%, 19.3%, and 2.5%, respectively.

Coded diagnosis and microbiologically defined cases were further compared to manual chart review for NTM disease. Coded diagnoses had a sensitivity of 42.9% (95%CI 12.8–71.5%), specificity of >99.9% (95%CI >99.9%), PPV of 38.2% (95%CI 15.8–73.4%), and NPV >99.9% (95%CI >99.9%) (including ICD-9-CM codes 031.8 and 031.9 resulted in a sensitivity of 97.0% but a PPV of 24.5%). Microbiology had a sensitivity of 84.6% (95%CI 54.6–100%), specificity of >99.9% (95%CI>99.9%), PPV of 68.0% (95%CI 45.9–90.1%), and NPV of >99.9% (95%CI >99.9%). When a composite diagnosis of either coded diagnosis or microbiology was allowed, we observed a sensitivity of 93.0% (95%CI 65.9–100%), specificity of >99.9% (95%CI >99.9%), PPV of 54.3% (95%CI 33.0–77.1%), and NPV>99.9% (95%CI >99.9%). During chart review, it was discovered that many veterans who had coded NTM diagnoses but lacked microbiology data had microbiologic studies that were performed and diagnoses made outside of VHA.

For subsequent analyses, we limited our analysis to calendar year 2009 and after so that the presence of each risk factor could be assessed for at least one year before the diagnosis of NTM. The incidence of NTM over the resulting time period was 12.6/100,000 patient-years. The 2012 national VA period prevalence of living patients with a history of NTM disease was 41.1 of every 100,000 live Veterans.

### Documentation of treatment

Of all patients that met criteria for pulmonary NTM disease, only 65.2% had records of VA-filled NTM-spectrum medications (either before or after the apparent diagnosis given that the initial diagnosis may not have been within VA) given more than 30 days. A somewhat lower proportion of those meeting criteria for extra-pulmonary NTM disease were given VA-filled medications (56.7%).

### Description of patients

The median age at diagnosis for both pulmonary and extra-pulmonary NTM cases was 65.1 years (Table 2). Almost all cases were among men: 96.7% of pulmonary and 95.9% of extra-pulmonary cases. The most common co-morbidity was COPD, which was found among 67.8% of pulmonary and 43.8% of extra-pulmonary cases. Most extra-pulmonary NTM cases were first recorded in the outpatient setting, while pulmonary cases were more mixed (77.0% outpatient extra-pulmonary and 48.4% outpatient pulmonary cases).

**Table 2:** NTM patient demographics and major co-morbidities, along with age- and setting-matched controls.

Characteristic	Pulmonary N = 2368	Control N = 9348	P	Extra-pulmonary N = 1222	Control N = 4868	p
Male	96.7%	96.0%	0.100	95.9%	94.3%	0.024
Age (median)	65.1	matched		65.1	Matched	
COPD	67.8%	37.8%	<0.001	43.8%	19.8%	<0.001
Bronchiectasis	7.0%	0.9%	<0.001	4.2%	0.4%	<0.001
Cancer	37.2%	29.6%	<0.001	30.1%	18.5%	<0.001
Other pulmonary	16.4%	8.8%	<0.001	9.9%	5.6%	<0.001
DMARD	4.8%	2.0%	<0.001	4.5%	1.4%	<0.001
HIV	6.4%	2.2%	<0.001	9.4%	1.7%	<0.001

COPD: Chronic obstructive pulmonary disease. DMARD: disease-modifying antirheumatic drugs, HIV: human immunodeficiency virus

### Geographic epidemiology

**Fig 2:**
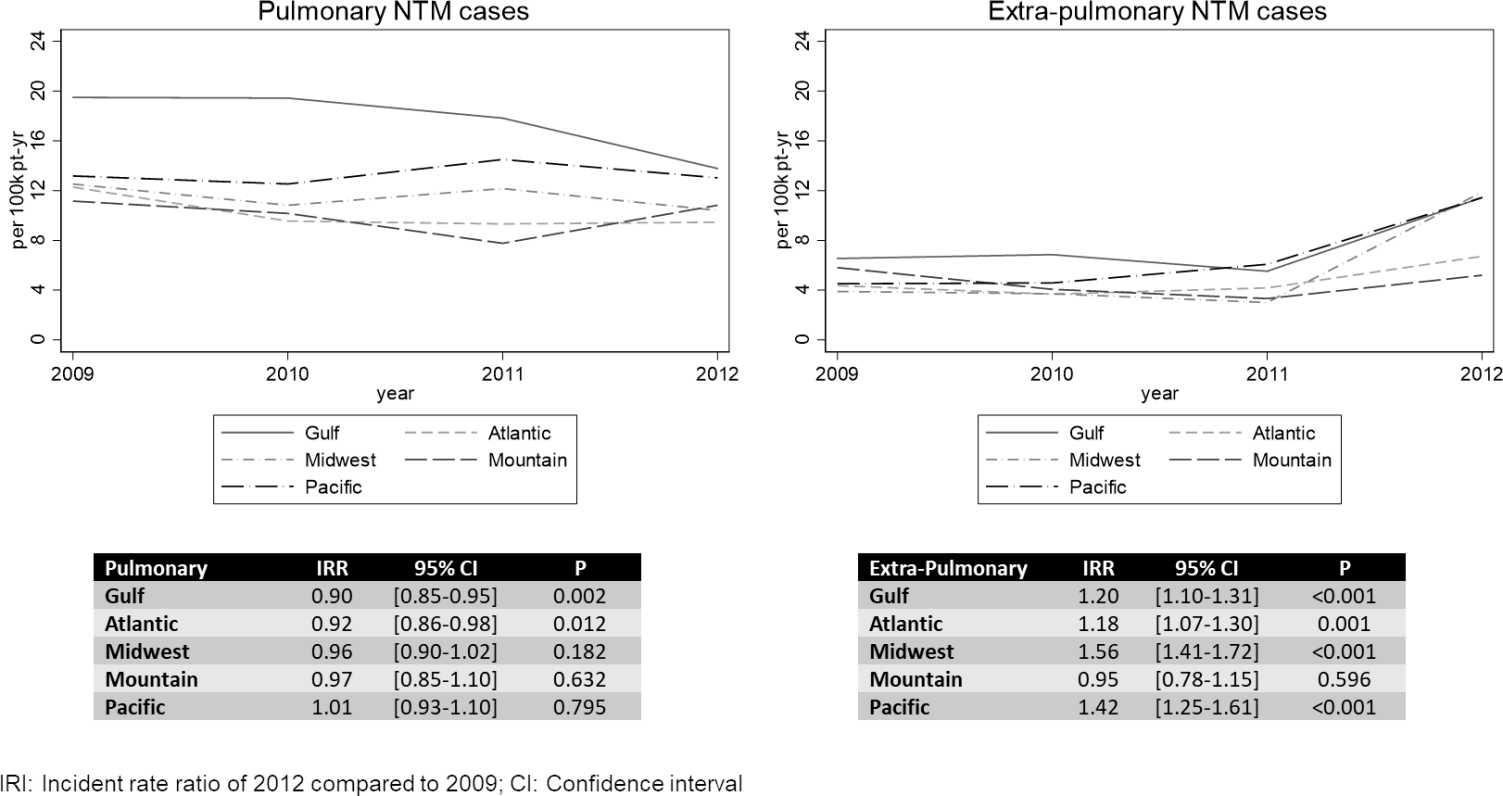
Incidence rates of NTM cases per 100,000 patient-years receiving care in VA over time.

**Fig 3:**
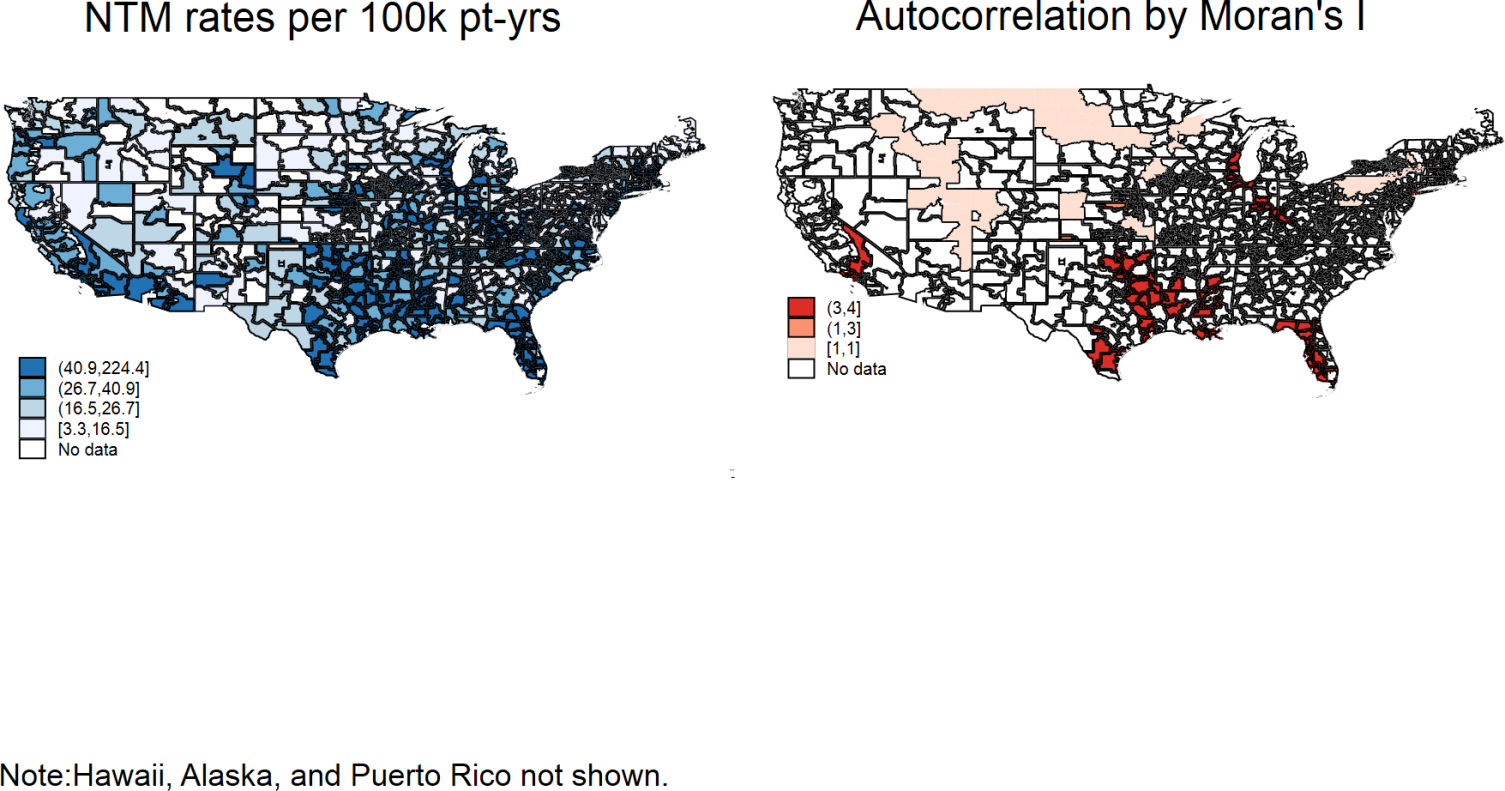
United States Veterans Health System regions grouped by first three digits of zip code. Incidence rates area denominated by 100,000 patient-years. Moran’s I measures the spatial correlation of a region with adjacent regions; positive correlations are >1, while negative correlations are <1. Reprinted from Esri^®^ ArcGIS Online^®^ under a CC BY license, with permission from Environmental Systems Research Institute, Inc., original copyright 2017.

Most NTM cases were from the Gulf Coast region, followed by the Midwest, Atlantic, Pacific, and lastly the Mountain regions (Figs 1–3). Although the Gulf Coast region had the most cases, there was a decreasing rate of pulmonary cases in that region (Fig 2). Significant overall downward trends in pulmonary incidence were also observed in the Atlantic and Gulf regions. In contrast, there were increases in overall extra-pulmonary disease in all but the Mountain region (Fig 2). The rates for *M*. *abscessus* group were higher in the Gulf Coast than in other regions most years (Fig 1).

Fig 3 demonstrates a finer map of the United States, according to the first 3 digits of zip codes. Some areas had higher NTM rates, particularly in the Southeastern U.S., Appalachia, and southern California and Arizona. Geographical clustering is indicated by spatial autocorrelation values in the same figure.

### Relationship to outcomes

**Fig 4:**
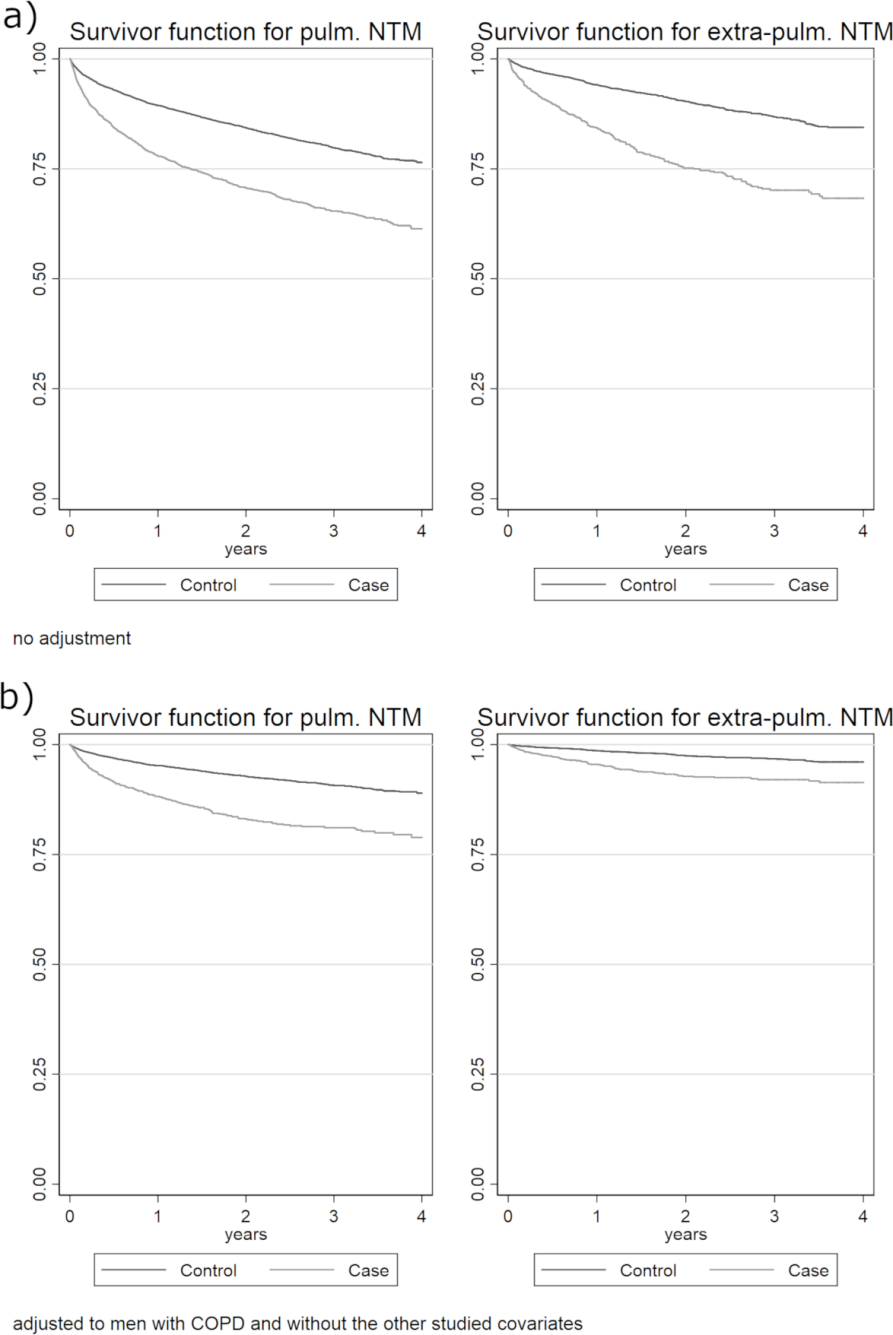
Kaplan-Meier curves of survival after pulmonary and extra-pulmonary NTM diagnosis compared to age- and clinical-setting matched controls.

Of 6,031 potential cases meeting either diagnostic or microbiologic criteria, 4,438 cases were found to have 4 appropriate matches; the rest were excluded. After exclusion of calendar year 2008 to allow for one year follow-up time for all remaining years, 3590 remained. The overall NTM disease incidence rate ratio of outpatient clinic visits in the first year after diagnosis was 1.34 (Table 3). Due to the violation of proportional hazards when analyzing mortality, piecewise models for early (<6 months) and late (> = 6 months) post-NTM detection periods were analyzed, wherein the assumption of proportional hazards was found to hold for each and each model contained roughly half of the outcomes. During the early period, NTM was associated with increased mortality (hazard ratio 1.4); however, in the late period, the association was stronger (hazard ratio 2.0). (Table 3 and Fig 4). To interpret the hazard ratios of the other covariates, we use COPD as an example. The hazard ratio of COPD in the late period was 1.39 when holding other covariates fixed within the matching strata.

**Table 3:** Multivariable models of outpatient clinic visit rates and mortality. Mortality is split into early and late periods in piecewise models.

Rate of outpatient clinic visits								
	IRR	95% CI		p				
NTM infection	1.34	1.34	1.35	<0.001				
Male	1.15	1.14	1.16	<0.001				
COPD	1.21	1.2	1.21	<0.001				
Bronchiectasis	0.89	0.88	0.9	<0.001				
Cancer	1.2	1.19	1.2	<0.001				
Other pulmonary dx	1.14	1.14	1.15	<0.001				
DMARD	1.38	1.37	1.39	<0.001				
HIV	0.98	0.97	0.99	<0.001				
Hazard of mortality	Early (<6 months)				Late (> = 6 months)			
	HR	95% CI		p	HR	95% CI		p
NTM infection	1.42	1.08	1.86	0.012	1.99	1.76	2.25	<0.001
Male	2.22	0.86	5.76	0.100	2.07	1.35	3.18	0.001
COPD	1.34	0.99	1.81	0.060	1.39	1.22	1.59	<0.001
Bronchiectasis	1.47	0.46	3.83	0.431	0.79	0.55	1.14	0.204
Cancer	1.3	0.99	1.71	0.063	1.48	1.31	1.68	<0.001
Other pulmonary dx	0.84	0.53	1.34	0.466	0.94	0.77	1.15	0.549
DMARD	0.99	0.45	2.21	0.990	1.59	1.13	2.24	0.008
HIV	0.75	0.28	1.97	0.554	1.24	0.87	1.8	0.221

COPD: Chronic obstructive pulmonary disease. DMARD: disease-modifying antirheumatic drugs, HIV: human immunodeficiency virus. Since included factors were identified *a priori* as suspected confounders, all factors were left in the model for adjustment.

## Discussion

The majority of NTM in VHA was found among older men with COPD. The Gulf Coast region had the highest rates of all NTM disease, especially that due to *M*. *abscessus*. The rate of extra-pulmonary NTM cases increased in most regions during the time period, in contrast to decreases in pulmonary NTM rates in multiple regions. NTM cases were independently associated with a higher hazard of death and rate of outpatient visits in the first year after diagnosis.

The geographic distribution of NTM disease in VHA was consistent with recent studies of Medicare beneficiaries [1, 3] and cystic fibrosis patients [23, 24], as well as with *M*. *avium* complex seroprevalence studies [12]. Our analysis, which included microbiology data is not strictly comparable to an independent VHA prevalence study of diagnostic codes but, geographically, there are similarities in distribution, as well as additional areas of interest found from microbiological data [25]. In a survey of 62 laboratories in 30 countries on 6 continents [26], *M*. *avium* complex was the most common (47%) overall, but ranged from 71% in Australia to 31% in South America (69.9% in our study).

Our finding of decreases in pulmonary NTM rates in multiple regions contrasts with other recent studies suggesting that pulmonary NTM is increasing in most settings. This may reflect changes in COPD prevalence in this population. The proportion of patients with COPD was high among our cases (67.8% of pulmonary and 43.8% of extrapulmonary cases) compared to controls (37.8% and 19.8%, respectively) and also compared to Veterans in general (7.8%) [16]. But there is some evidence that COPD prevalence may be peaking in reports from non-VA settings [27]. In contrast, the rate of extra-pulmonary NTM cases increased in most regions during our study period. This may be due to an increasing amount of immunosuppression in our population; however, data on immunosuppression trends are currently lacking and a visual inspection of the upward trend suggests that it is restricted to 2012 [28].

Our understanding of NTM impact has improved recently due to the availability of larger data sets [3–5, 8, 29] and heightened awareness from higher mortality among immunocompromised populations [30]. A recent sample of US Medicare and Medicaid data demonstrated an OR of 1.4 for death during the study compared to non-cases [1], consistent with our findings but subject to ICD-9-CM code specificity problems detailed below. NTM is also linked with higher morbidity. A Taiwanese study showed a 4-fold higher odds of respiratory failure among those with NTM [29], a finding that may help explain the higher burden of care (more outpatient clinic visits) observed in our population.

The strengths of this study are its size and the novel data extraction methods used to retrieve data. Limitations to this study include ascertainment error; we know that some patients may be diagnosed with NTM disease outside of VHA but have continuing care in the system. This likely led to a bias toward late identification of incident cases diagnosed outside of VHA, as well as some missed cases. A longer period of observation drawing from complete medical records (including care outside VHA) prior to each case would likely have led to less bias toward late identification and less misclassification of individuals as incident when they were prevalent. Measurement of period prevalence would have been less biased by late cases. However, we would have had to assume that NTM impact was for life and would have observed exaggerated effects of false positives on our subsequent analyses (where inclusion of false positives may live longer than true cases and introduce a bias toward the null). This potential for error can affect all of our estimates. Despite high specificity, the positive predictive value of our combined microbiology and diagnosis code case definition was not high, due to low prevalence of NTM in the population, although microbiology alone was high and consistent with that seen in other settings [31]. The ICD-9-CM and microbiology components of our definition demonstrated considerable disagreement. Although we validated our algorithms, it was not feasible to review every case to confirm the diagnosis, thus likely introducing a bias toward the null in our analyses. The imperfect (~60%) proportion of treatment among identified cases suggests either a fair amount of antibiotic prescription outside of VHA or that the specificity of our electronic case definition is low. However, in other studies, only about a third of confirmed cases received NTM antimicrobial therapy [31]. Conversely, our definition of NTM-spectrum therapy is not specific and may overestimate treatment. Although this finding merits further exploration, it is interesting that this proportion of cases receiving treatment is close to our measured PPV. There may also be misclassification between pulmonary and extra-pulmonary cases. There is also undoubtedly residual confounding. Therefore, we may only conclude that NTM is associated with clinic utilization and mortality.

In summary, there is evidence that NTM disease is associated with considerable morbidity and mortality in VHA. The rising rate of extra-pulmonary NTM will require further study and follow-up. To gain a comprehensive understanding of NTM epidemiology within a health system, it is likely necessary to examine both diagnosis and microbiology data. Because of our poor understanding of NTM, it may be appropriate to develop policies and procedures to minimize the risks of macrolide monotherapy in severe COPD patients in VHA, e.g., possibly by periodic screening of sputa specimens for NTM. Future studies investigating the relationships between NTM disease, patient outcomes, and healthcare utilization have the potential to improve NTM treatment models, e.g., by establishing or reinforcing regional consultation programs.

## References

[1] Adjemian J, Olivier KN, Seitz AE, Holland SM, Prevots DR. Prevalence of nontuberculous mycobacterial lung disease in U.S. Medicare beneficiaries. American journal of respiratory and critical care medicine. 2012;185(8):881–6. doi: 10.1164/rccm.201111-2016OC 22312016 10.1164/rccm.201111-2016OC PMC3360574

[2] Billinger ME, Olivier KN, Viboud C, de Oca RM, Steiner C, Holland SM, et al Nontuberculous mycobacteria-associated lung disease in hospitalized persons, United States, 1998–2005. Emerging infectious diseases. 2009;15(10):1562–9. doi: 10.3201/eid1510.090196 19861046 10.3201/eid1510.090196 PMC2866394

[3] Adjemian J, Olivier KN, Seitz AE, Falkinham JO 3rd, Holland SM, Prevots DR. Spatial clusters of nontuberculous mycobacterial lung disease in the United States. American journal of respiratory and critical care medicine. 2012;186(6):553–8. doi: 10.1164/rccm.201205-0913OC 22773732 10.1164/rccm.201205-0913OC PMC3480533

[4] Martin-Casabona N, Bahrmand AR, Bennedsen J, Thomsen VO, Curcio M, Fauville-Dufaux M, et al Non-tuberculous mycobacteria: patterns of isolation. A multi-country retrospective survey. Int J Tuberc Lung Dis. 2004;8(10):1186–93. 15527150

[5] Moore JE, Kruijshaar ME, Ormerod LP, Drobniewski F, Abubakar I. Increasing reports of non-tuberculous mycobacteria in England, Wales and Northern Ireland, 1995–2006. BMC Public Health. 2010;10:612 doi: 10.1186/1471-2458-10-612 20950421 10.1186/1471-2458-10-612 PMC2964631

[6] Jing H, Wang H, Wang Y, Deng Y, Li X, Liu Z, et al Prevalence of nontuberculous mycobacteria infection, China, 2004–2009. Emerging infectious diseases. 2012;18(3):527–8. doi: 10.3201/eid1803.110175 22376989 10.3201/eid1803.110175 PMC3309567

[7] du Moulin GC, Sherman IH, Hoaglin DC, Stottmeier KD. Mycobacterium avium complex, an emerging pathogen in Massachusetts. J Clin Microbiol. 1985;22(1):9–12. 3874880 10.1128/jcm.22.1.9-12.1985 PMC268310

[8] Mirsaeidi M, Machado RF, Garcia JG, Schraufnagel DE. Nontuberculous mycobacterial disease mortality in the United States, 1999–2010: a population-based comparative study. PloS one. 2014;9(3):e91879 doi: 10.1371/journal.pone.0091879 24632814 10.1371/journal.pone.0091879 PMC3954860

[9] Al-Houqani M, Jamieson F, Mehta M, Chedore P, May K, Marras TK. Aging, COPD, and other risk factors do not explain the increased prevalence of pulmonary Mycobacterium avium complex in Ontario. Chest. 2012;141(1):190–7. doi: 10.1378/chest.11-0089 21724552 10.1378/chest.11-0089

[10] Marras TK, Mehta M, Chedore P, May K, Al Houqani M, Jamieson F. Nontuberculous mycobacterial lung infections in Ontario, Canada: clinical and microbiological characteristics. Lung. 2010;188(4):289–99. doi: 10.1007/s00408-010-9241-8 20383715 10.1007/s00408-010-9241-8

[11] Donohue MJ, Mistry JH, Donohue JM, O'Connell K, King D, Byran J, et al Increased Frequency of Nontuberculous Mycobacteria Detection at Potable Water Taps within the United States. Environ Sci Technol. 2015;49(10):6127–33. doi: 10.1021/acs.est.5b00496 25902261 10.1021/acs.est.5b00496

[12] Khan K, Wang J, Marras TK. Nontuberculous mycobacterial sensitization in the United States: national trends over three decades. American journal of respiratory and critical care medicine. 2007;176(3):306–13. doi: 10.1164/rccm.200702-201OC 17507546 10.1164/rccm.200702-201OC

[13] Albert RK, Connett J, Bailey WC, Casaburi R, Cooper JA Jr., Criner GJ, et al Azithromycin for prevention of exacerbations of COPD. The New England journal of medicine. 2011;365(8):689–98. doi: 10.1056/NEJMoa1104623 21864166 10.1056/NEJMoa1104623 PMC3220999

[14] Fennelly KP, Griffith DE. Azithromycin in non-cystic-fibrosis bronchiectasis. Lancet. 2013;381(9860):27. 10.1016/S0140-6736(13)60013-6 23290963

[15] Murphy DE, Chaudhry Z, Almoosa KF, Panos RJ. High prevalence of chronic obstructive pulmonary disease among veterans in the urban midwest. Military medicine. 2011;176(5):552–60. 21634301 10.7205/milmed-d-10-00377

[16] Finkelstein J, Cha E, editors. Association of Veteran Status with COPD Prevalence Stratified by Gender. American Thoracic Society International Conference; 2013; Pennsylvania Convention Center: American Journal of Respiratory and Critical Care Medicine.

[17] Ford ES, Croft JB, Mannino DM, Wheaton AG, Zhang X, Giles WH. COPD surveillance—United States, 1999–2011. Chest. 2013;144(1):284–305. doi: 10.1378/chest.13-0809 23619732 10.1378/chest.13-0809 PMC3707177

[18] Patterson O, Igo S, Hurdle JF. Automatic acquisition of sublanguage semantic schema: towards the word sense disambiguation of clinical narratives. AMIA Annu Symp Proc. 2010;2010:612–6. 21347051 PMC3041300

[19] Griffith DE, Aksamit T, Brown-Elliott BA, Catanzaro A, Daley C, Gordin F, et al An official ATS/IDSA statement: diagnosis, treatment, and prevention of nontuberculous mycobacterial diseases. American journal of respiratory and critical care medicine. 2007;175(4):367–416. doi: 10.1164/rccm.200604-571ST 17277290 10.1164/rccm.200604-571ST

[20] Carpenter J, Bithell J. Bootstrap confidence intervals: when, which, what? A practical guide for medical statisticians. Statistics in medicine. 2000;19(9):1141–64. 10797513 10.1002/(sici)1097-0258(20000515)19:9<1141::aid-sim479>3.0.co;2-f

[21] Pepe M. The Statistical Evaluation of Medical Tests for Classification and Prediction New York: Oxford University Press; 2003.

[22] Pisati M. Exploratory spatial data analysis using Stata. German Stata Users' Group Meetings 20122012.

[23] Prevots DR, Adjemian J, Fernandez AG, Knowles MR, Olivier KN. Environmental risks for nontuberculous mycobacteria. Individual exposures and climatic factors in the cystic fibrosis population. Annals of the American Thoracic Society. 2014;11(7):1032–8. doi: 10.1513/AnnalsATS.201404-184OC 25068620 10.1513/AnnalsATS.201404-184OC PMC4214058

[24] Adjemian J, Olivier KN, Prevots DR. Nontuberculous mycobacteria among patients with cystic fibrosis in the United States: screening practices and environmental risk. American journal of respiratory and critical care medicine. 2014;190(5):581–6. doi: 10.1164/rccm.201405-0884OC 25068291 10.1164/rccm.201405-0884OC PMC4214089

[25] Schweitzer M, Bagley V, Balestrini K, Guerrero A, Sharifi A, Campos A, et al, editors. Non-Tuberculous Mycobacteria in Chronic Obstructive Pulmonary Disease American Thoracic Society 2017; 2017; Washington, D.C.

[26] Hoefsloot W, van Ingen J, Andrejak C, Angeby K, Bauriaud R, Bemer P, et al The geographic diversity of nontuberculous mycobacteria isolated from pulmonary samples: an NTM-NET collaborative study. The European respiratory journal. 2013;42(6):1604–13. doi: 10.1183/09031936.00149212 23598956 10.1183/09031936.00149212

[27] Simpson CR, Hippisley-Cox J, Sheikh A. Trends in the epidemiology of chronic obstructive pulmonary disease in England: a national study of 51 804 patients. Br J Gen Pract. 2010;60(576):277–84. doi: 10.3399/bjgp10X514729 20594429 10.3399/bjgp10X514729 PMC2894402

[28] Harpaz R, Dahl RM, Dooling KL. Prevalence of Immunosuppression Among US Adults, 2013. JAMA. 2016;316(23):2547–8. doi: 10.1001/jama.2016.16477 27792809 10.1001/jama.2016.16477

[29] Yeh JJ, Wang YC, Sung FC, Chou CY, Kao CH. Nontuberculosis mycobacterium disease is a risk factor for chronic obstructive pulmonary disease: a nationwide cohort study. Lung. 2014;192(3):403–11. doi: 10.1007/s00408-014-9574-9 24691889 10.1007/s00408-014-9574-9

[30] Huang HC, Weigt SS, Derhovanessian A, Palchevskiy V, Ardehali A, Saggar R, et al Non-tuberculous mycobacterium infection after lung transplantation is associated with increased mortality. The Journal of heart and lung transplantation: the official publication of the International Society for Heart Transplantation. 2011;30(7):790–8. 10.1016/j.healun.2011.02.007 PMC3942162 21482148

[31] Winthrop KL, McNelley E, Kendall B, Marshall-Olson A, Morris C, Cassidy M, et al Pulmonary nontuberculous mycobacterial disease prevalence and clinical features: an emerging public health disease. American journal of respiratory and critical care medicine. 2010;182(7):977–82. doi: 10.1164/rccm.201003-0503OC 20508209 10.1164/rccm.201003-0503OC

